# Peripheral CD56^+^CD16^+^ NK Cell Populations in the Early Follicular Phase Are Associated With Successful Clinical Outcomes of Intravenous Immunoglobulin Treatment in Women With Repeated Implantation Failure

**DOI:** 10.3389/fendo.2019.00937

**Published:** 2020-01-21

**Authors:** Yao-Kai Ho, Hsiu-Hui Chen, Chun-Chia Huang, Chun-I Lee, Pin-Yao Lin, Maw-Sheng Lee, Tsung-Hsien Lee

**Affiliations:** ^1^Institute of Medicine, Chung Shan Medical University, Taichung, Taiwan; ^2^Department of Obstetrics and Gynecology, Chung Shan Medical University Hospital, Taichung, Taiwan; ^3^Division of Infertility, Lee Women's Hospital, Taichung, Taiwan

**Keywords:** natural killer cells, intravenous immunoglobulin, repeated implantation failure, infertility, lymphocytes

## Abstract

The percentage of peripheral CD56^+^CD16^+^ NK cells in the early follicular phase on days 2–3 of the menstrual cycle in repeated implantation failure (RIF) patients was used to evaluate the impact of intravenous immunoglobulin (IVIG) on ART cycles. A total 283 patients with RIF consisting of at least 3 ART failures and at least 2 high quality embryo transfers were recruited. A logistic regression analysis for the peripheral immunological profile was completed to predict implantation success and compare the implantation and pregnancy rates between groups with ≤10.6 and >10.6% of CD56^+^CD16^+^ NK cells in the early follicular phase. The logistic regression and receiving operating curve analyses showed that patients with ≤ 10.6% of peripheral CD56^+^CD16^+^ NK cells in the early follicular phase showed a lower pregnancy rate within the RIF group without IVIG. Patients with peripheral CD56^+^CD16^+^ NK cells ≤ 10.6% and without IVIG treatment showed significantly lower implantation and pregnancy rates (12.3 and 30.3%, respectively) when compared with the CD56^+^CD16^+^ NK cells >10.6% group (24.9 and 48.0%, respectively, *p* < 0.05). Furthermore, the patients with CD56^+^CD16^+^ NK cells ≤ 10.6% given IVIG starting before ET had significantly higher implantation, pregnancy, and live birth rates (27.5, 57.4, and 45.6%, respectively) when compared with the non-IVIG group (12.3, 30.3, and 22.7%, respectively, *p* < 0.05). Our results showed that a low percentage of peripheral CD56^+^CD16^+^ NK cells (≤10.6%) in the early follicular phase is a potential indicator of reduced pregnancy and implantation success rates in RIF patients, and IVIG treatment will likely benefit this patient subgroup.

## Introduction

With advances in assisted reproduction techniques (ART), high quality embryos can be imbedded into the uterus for pregnancy. However, a substantial number of women suffer from the repeated implantation failure (RIF) of several embryos, regardless of quality (1). For many years, defective crosstalk between the embryo and endometrium in unexplained RIF patients was attributed to circulating peripheral blood mononuclear cells (PBMC) and immunological responses, with the exception of inherent genetic, anatomical, chromosomal, or endocrine abnormalities (2). Fujiwara et al. suggest that circulating blood cells positively contribute to maternal tissue remodeling (2).

Pregnancy evolves through different immunological stages with a pro-inflammatory or anti-inflammatory predominant profile, depending on the stage of gestation analyzed (3, 4). Increasing evidence indicates that immune cell or immunologic factors play an important role in the failure of both natural and ART-induced pregnancies (3–5). Monocyte/macrophage lineage cell markers increase in the decidua/myometrium during pregnancy and may control trophoblast cell invasion into the myometrium while preventing a rejection of the semi-allogenic conceptus to provide an important barrier against invading pathogens (5, 6).

For RIF patients, there are beneficial effects of intravenous immunoglobulin (IVIG) purified from the pooled blood plasma of healthy donors (7, 8). The proposed mechanisms of action of IVIG are categorized into direct antibody effects (9) and immune-modulation (10). Furthermore, preconception immune testing from peripheral blood may be a critical tool for determining which patients will benefit from IVIG therapy (7). Measurements of peripheral blood immune cells by flow cytometry are easier and less invasive because this technique does not require obtaining an endometrial biopsy sample.

Jurisic et al. in 2007 reported that increase in the concentration of IgG immunoglobulins significantly correlated with increase of NK cell activity (11). NK cells constitute 5–10% of peripheral blood lymphocytes (PBL) and have a CD3^−^CD16^+^ CD56^+^ phenotype (12). NK cells play an important role in cancer (11, 12), viral infections and gynecology (13, 14), transplantation immunology (15), especially because they are cells of innate immunity. Furthermore, natural killer cells play an essential role in defense of the rise and spread of malignancy (11). The multiple myeloma patients with higher NK cell activity at presentation have better cumulative survival in comparison with those with low NK cell activity.

Previous studies indicated that the percentage of peripheral blood NK cells in the luteal phase were significantly increased in women with recurrent pregnancy losses or implantation failures (16, 17). However, a systematic review by Tang et al. reported that the prognostic value of measuring pNK or uNK cell parameters remains unclear (18), and more studies are needed to confirm or refute the role of NK cell assessments as a predictive test for screening in recurrent miscarriage (RM) or RIF patients. Therefore, the relevance between peripheral mononuclear profiles and RIF deserves further investigation. In the present study, peripheral blood monocytes (PBMC) samples were collected from RIF women in the early follicular phase instead of the luteal phase. We compared the PBMC profile in the early follicular phase with controlled ovarian stimulation and IVF outcomes after IVIG in RIF patients to identify candidates for IVIG treatment.

## Materials and Methods

### Patient Selection

The entire study population was comprised of 283 women with RIF who were referred to the Lee Women's Hospital and treated with *in vitro* fertilization (IVF) protocols between Jan. 2007 and Oct. 2011. This study consisted of Human Subject Research. The study protocol was approved by the Institutional Review Board of the Chung Shan Medical University Hospital (CSMUN No. CS:12033). All participants provided their written informed consent to participate in this study; in addition, all participants signed standard IVF consent forms. The written consents of IVIG treatment were obtained from journal meeting records or patient treatment charts in the administration department at Lee's Women Hospital. The journal meetings or consultations in the IVF laboratory at Lee's Women Hospital were held every week, and all participants signed a consent form after the meeting. At least one signature of each participant was recorded during study. Written consent was not obtained from patients in these meetings who were not associated this study or participated in other unpublished studies. The ethics committees/IRBs approved this consent procedure, and the invasion of patient privacy was avoided in this study. All patients were recruited based upon a history of repeat implantation failure with unknown reasons. After delicate counseling, we provided IVIG treatment as an alternative strategy for the possible immune reasons. The choice of IVIG treatment was dependent on the couples. Patients who decided to receive IVIG therapy signed an IVIG consent form that explained the possible risks, the nature of the medication, and the lack of sufficient evidence-proof for treatment efficacy. Inclusion criteria of RIF patients in this study included patients who experienced >2 failures of IVF–embryo transfer therapy with at least two good embryos transferred each session. The following exclusion criteria were used for this study: (i) abnormal uterine anatomy evaluated by hysterosalpingography and /or hysteroscopy; (ii) abnormal blood karyotype in the female or male partner; (iii) positive titer for the lupus anticoagulant; (iv) endometriosis; (v) recurrent miscarriage; (vi) endometrium ≤ 7 mm on the day of hCG injection; or (vii) BMI≥30.

### IVF Protocol

All women underwent a program consisting of a long protocol for GnRH agonist administration (19). Participating women were administered leuprolide acetate (Lupron, Takeda Chemical Industries, Ltd., Osaka, Japan) starting at the midluteal phase to produce down-regulation. All patients subsequently received recombinant follicular stimulation hormone (rFSH; Gonal-F, Serono, Bari, Italy) for ovarian stimulation from cycle day 3 until the dominant follicle reached a diameter of >18 mm. Next, patients received an injection of 250 micrograms of human chorionic gonadotropin (hCG; Ovidriell, Serono) 36 h prior to oocyte retrieval.

### IVIG Treatment Protocol

**Figure 1:**
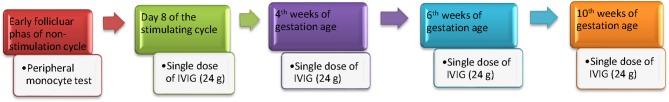
The timing and protocol of IVIG treatment. Peripheral monocyte test was performed on the 2–3rd day of the menstrual cycle prior to ovarian hyperstimulation. Women received the first dose of IVIG (24 g TBSF human immunoglobulin; CSL Limited, Broadmeadous, Australia) on day 8 of the stimulating cycle. If a viable pregnancy was confirmed by serum hCG concentrations and ultrasound, IVIG was continued in 4, 6, and 10th weeks of gestation age (a total dose of 96 g) according to the published protocol.

The IVF and IVIG treatment protocols are shown in Figure 1. Patients received the first dose of IVIG (24 g TBSF human immunoglobulin; CSL Limited, Broadmeadous, Australia) on day 8 of the stimulating cycle. If a viable pregnancy was confirmed by serum hCG concentrations and ultrasound, IVIG was continued in the 4, 6, and 10th weeks of gestation age (a total dose of 96 g) according to the published protocol (20). Patients in the non-IVIG treatment group did not receive a placebo treatment during stimulation and pregnancy.

### Embryo Culture

After retrieval, oocytes were cultured in Quinn's Advantage Fertilization Medium (Sage BioPharma, Inc., Trumbull, CT, USA) with a 10% serum protein substitute (SPS, Sage BioPharma, Inc) in a triple gas phase of 5% CO_2_, 5% O_2_, and 90% N_2_. Following conventional insemination or ICSI, all embryos were furthered cultured in microdrops of cleavage medium (Sage BioPharma, Inc., Trumbull, CT, USA) with a 10% serum protein substitute. Fertilization was verified by the presence of two pronuclei 17–19 h after insemination or injection. The embryo transfer was performed on day 3. Embryos with the most favorable cell number, fragmentation, and asymmetry scores were selected for transfer. Each patient's age, history, and number and morphology of available embryos were utilized to determine the number of embryos to transfer. Clinical pregnancies were diagnosed by the presence of a gestational sac on transvaginal ultrasound 5 weeks after oocyte retrieval.

### Peripheral Blood Test and Flow Cytometry

**Figure 2:**
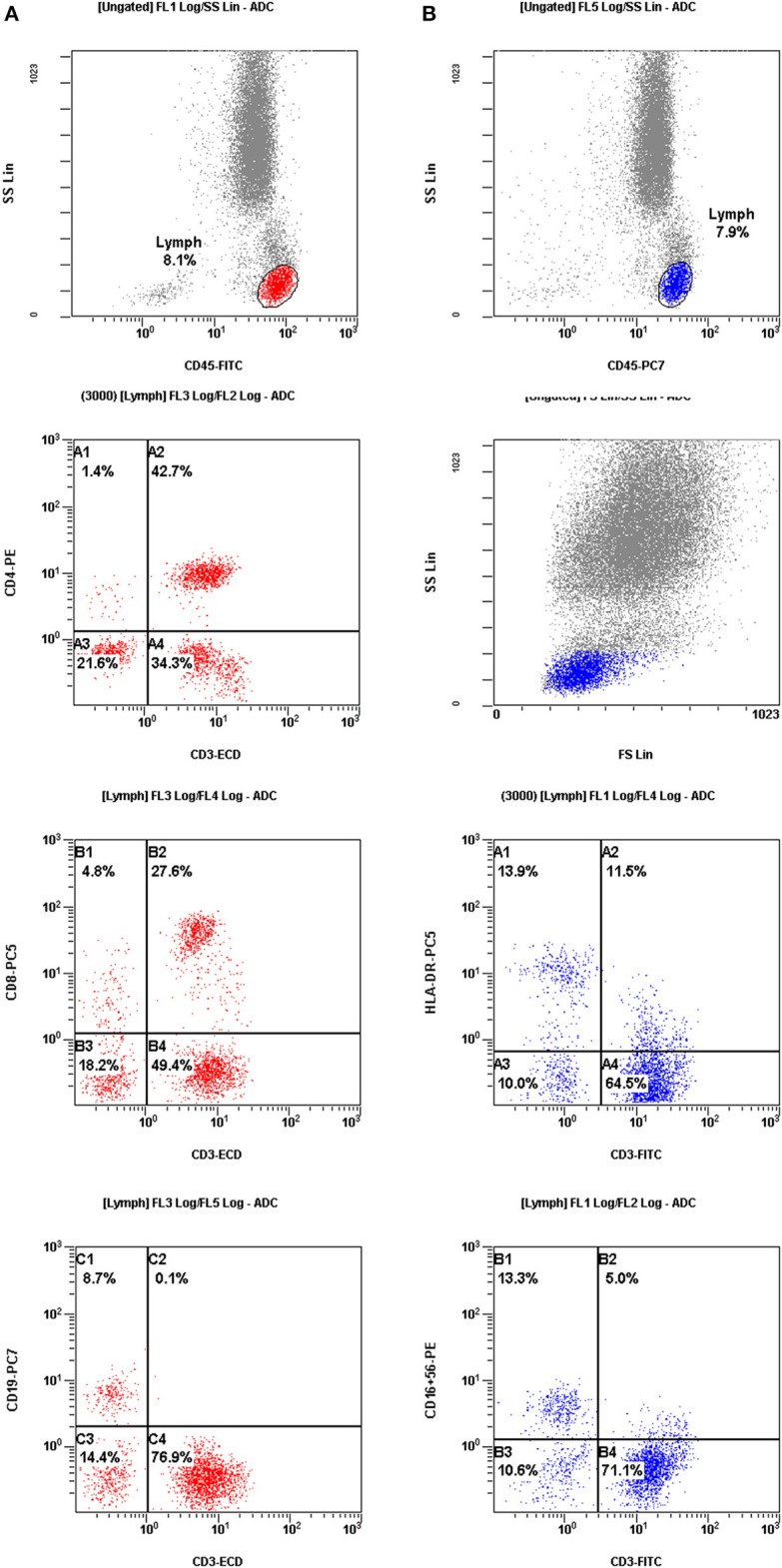
The results were presented as a percentage of total lymphocytes. Cell sorting for assessment of the mononuclear cell profiles in peripheral blood from patients with repeated implantation failure (RIF). **(A)** (red color) Mononuclear cells were gated by fluorescence intensity of CD45 vs. side light scatter (SSL). The T cells (CD3^+^, C4) and B cells (CD19^+^, C1) were gated by fluorescence intensity of CD3 vs. CD19. The helper T cells (CD3^+^CD4^+^, A2) were analyzed for their expression of CD3 and CD4. The suppressor T cells (CD3^+^CD8^+^, B2) were gated by fluorescence intensity of CD3 vs. CD8. **(B)** (blue color) Activated T cells (CD3^+^ HLA-DR^+^, A2) were analyzed by the expression of CD3 and HLA-DR. CD56+CD16+ NK cells (CD3^−^CD56^+^CD16 ^+^, B1) were gated by fluorescence intensity of CD3 versus CD16^+^CD56^+^.

The peripheral blood of RIF women was sampled simultaneously on days 2–3 of the menstrual cycle prior to ovarian hyperstimulation. All of the blood samples from RIF patients were tested for the presence of autoantibodies, such as lupus anticoagulant (LA), anti-cardiolipin antibodies and antinuclear antibodies (ANA). No patients had any type of infection during the last month before blood collection. In the same blood sample, the PBMC profile was determined using flow cytometry. Three-color flow cytometry (FACS Calibur, BD Biosciences) was used to evaluate different mononuclear cell subpopulations. The examination was completed using fresh blood prior to the IVF procedure. The differently labeled monoclonal antibodies were used in following combinations: CD3^+^, CD4^+^, CD8^+^, CD19^+^, CD-AT (activated T cell; CD3^+^/HLA-DR^+^) and NK (CD16^+^CD56^+^). All of the reagents were produced by Becton Dickinson (BD Biosciences, Franklin Lakes, NJ, USA). The results are presented as a percentage of total lymphocytes (Figure 2).

### Statistical Analysis

Differences between the IVIG and non-IVIG (control) groups with regard to age, numbers of embryo transfer attempts, numbers of transferred embryos, and numbers of good quality embryos were analyzed by Student's *t*-test. A Chi-square test was used for comparisons of the clinical pregnancy rate, implantation rate and live birth rate between groups. A *P* < 0.05 was considered significant. All calculations were performed using SPSS 17.0 (StatSoft Inc., Tulsa, USA) and MedCalc Statistical Software version 12.7.7 (MedCalc Software bvba, Ostend, Belgium; 2013).

## Results

Table 1 shows the demographic data and treatment outcomes for the control and IVIG groups. There was a total of 283 completed treatment cycles comprised of 115 cycles in the IVIG group and 168 cycles in the control (non-IVIG) group. The mean of age, etiology of infertility, previous IVF times, and embryo transferred numbers between IVIG and non-IVIG groups were not significantly different. The implantation [(26.5%, 108/408) vs. (20.1%, 111/553)] and pregnancy [(59.1%, 68/115) vs. (41.1%, 69/168)] rates in the IVIG group were significantly higher when compared with the non-IVIG group. The live birth rate [(30.4%, 51/168) vs. (43.5%, 50/1,150; *p* = 0.079)] in the IVIG group showed a non-significant trend toward an increase when compared with the non-IVIG group. The abortion rates, fetal body weight and gestational age of birth were not significantly different between the two groups.

**Table 1:** Demographic data of the control (Non-IVIG) and IVIG treatment groups.

	**Control (*n* = 168)**	**IVIG (*n* = 115)**	P^*^
Age (years)	36.5 ± 4.4	35.4 ± 4.7	0.082
BMI (Kg/m^2^)	21.4 ± 3.1	21.3 ± 2.7	0.254
**Infertility**			
Male factor (%)	31.5 (53/168)	32.3 (44/115)	0.887
Female factor (%)	39.3 (66/168)	31.3 (36/115)	0.169
Combined factor (%)	8.9 (15/168)	10.4 (12/115)	0.673
Unexplained (%)	20.2 (34/168)	20.0 (23/115)	0.967
Previous IVF times	5.1 ± 2.4	5.4 ± 2.9	0.244
Oocyte number	14.7 ± 9.5	15.3 ± 10.6	0.579
MII number	11.8 ± 7.8	12.3 ± 8.5	0.606
Fertilized embryo number	9.5 ± 6.6	9.9 ± 7.0	0.589
High qualified embryo rate	72.1 ± 14.0	70.0 ± 12.6	0.201
Transferred embryos	3.3 ± 0.8	3.5 ± 0.6	0.078
Implantation rate (%)	20.1 (111/553)	26.5 (108/408)	0.019
Pregnancy rate (%)	41.1 (69/168)	59.1 (68/115)	0.003
Live birth rate (%)	30.4 (51/168)	43.5 (50/115)	0.079
Abortion rate (%)	24.6 (17/69)	25.0 (17/68)	0.957
Fetal body weight (g)	2,710 ± 622	2,489 ± 619	0.083
Gestational age of delivery (weeks)	36.8 ± 2.8	36.2 ± 2.5	0.254

* *Comparison by Mann Whitney U test or X^2^ test as the condition determined*.

*The data are presented with mean± standard deviation (SD) or percentage (%)*.

No adverse effects related to the infusion occurred in the IVIG group. None of the patients discontinued IVIG therapy because of side effects, and the IVIG treatment did not produce significant toxicity in the mother or fetus.

### The Percentage of Peripheral CD56^+^CD16^+^ NK Cell in the Early Follicle Phase May Predict the Art Outcome

A logistic regression analysis of the control group was completed to define the correlation between the peripheral mononuclear cell profile and pregnancy (at least one embryo implantation) outcome of IVF treatment. When a single variable in the peripheral monocyte profile was used in the analysis model, both CD19 and CD56^+^CD16^+^ NK cell percentages demonstrated a significant correlation with ART pregnancy outcome (Table 2).

**Table 2:** Logistic regression analysis for the peripheral immunological profile to predict pregnancy (implantation success) by control group (Non-IVIG treatment, *n* = 168).

	**Mono-variable**	
	**Regression coefficient (95% CI)**	***p***
CD3	0.976 (0.940–1.014)	0.208
CD4	0.986 (0.947–1.026)	0.474
CD8	0.997 (0.956–1.039)	0.874
CDAT	1.078 (0.995–1.168)	0.065
CD19	0.922 (0.859–0.990)	0.026
NK	1.071 (1.021–1.124)	0.005

**Figure 3:**
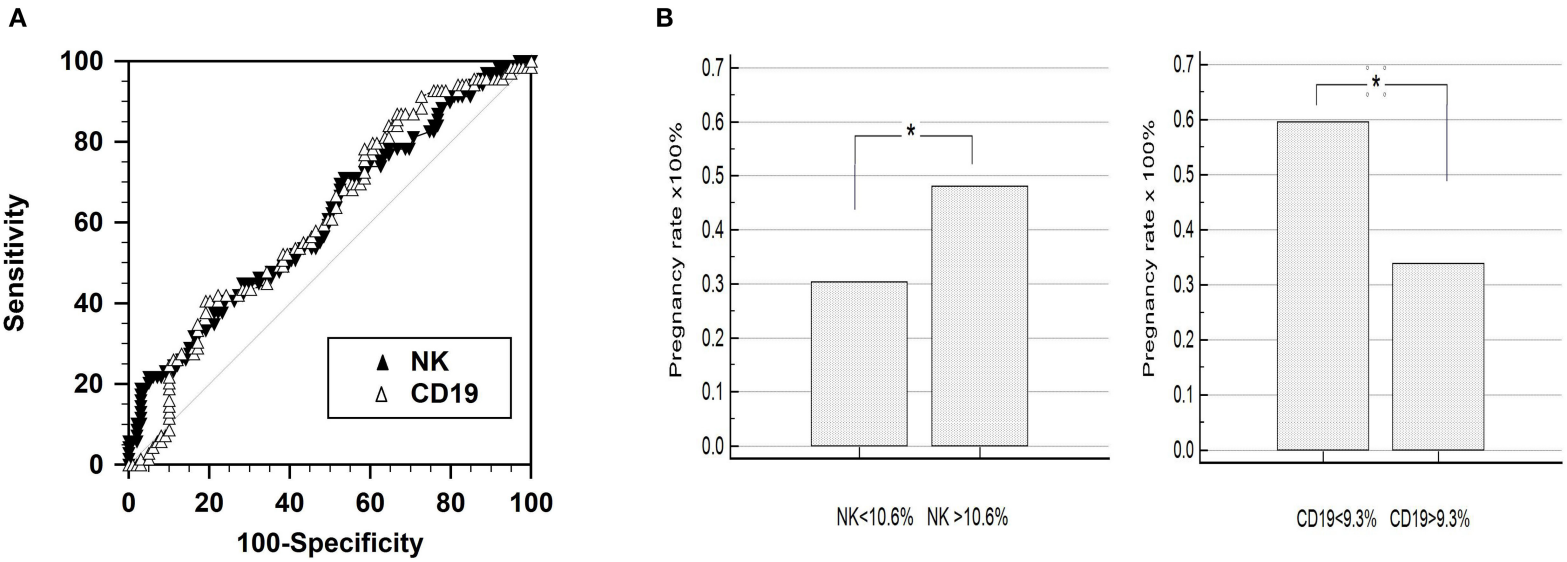
Receiver operating characteristic (ROC) curve analysis of CD56+CD16+ NK cell percentage. **(A)** Comparison of the predictive value for pregnancy outcome by means of area under the curve of receiver operating characteristic (ROC_AUC_) curve analysis. The percentage of peripheral natural killer (NK; CD56+CD16+) and B (CD19) cells featured similar ROC_AUC_ for ART outcome. **(B)** Pregnancy outcome of the groups divided by the criteria selected by the ROC curve analysis: 10.6% for the NK cells (CD3-CD56+CD16+) and 9.3% for the B cells (CD19+), respectively.

A receiver operating characteristic (ROC) curve analysis revealed the cut-off value for pregnancy outcome was 10.6% of CD56^+^CD16^+^ NK cells and 9.3% of CD19^+^ B cells (Figures 3A,B). This selected value of NK cells had a sensitivity of 71.0% (95% CI: 58.8–81.3) and a specificity of 46.5% (95% CI: 36.4–56.8). The selected values of B cells had a sensitivity of 40.6% (95% CI: 28.9–53.1) and a specificity of 80.8% (95% CI: 71.7–88.0).

**Figure 4:**
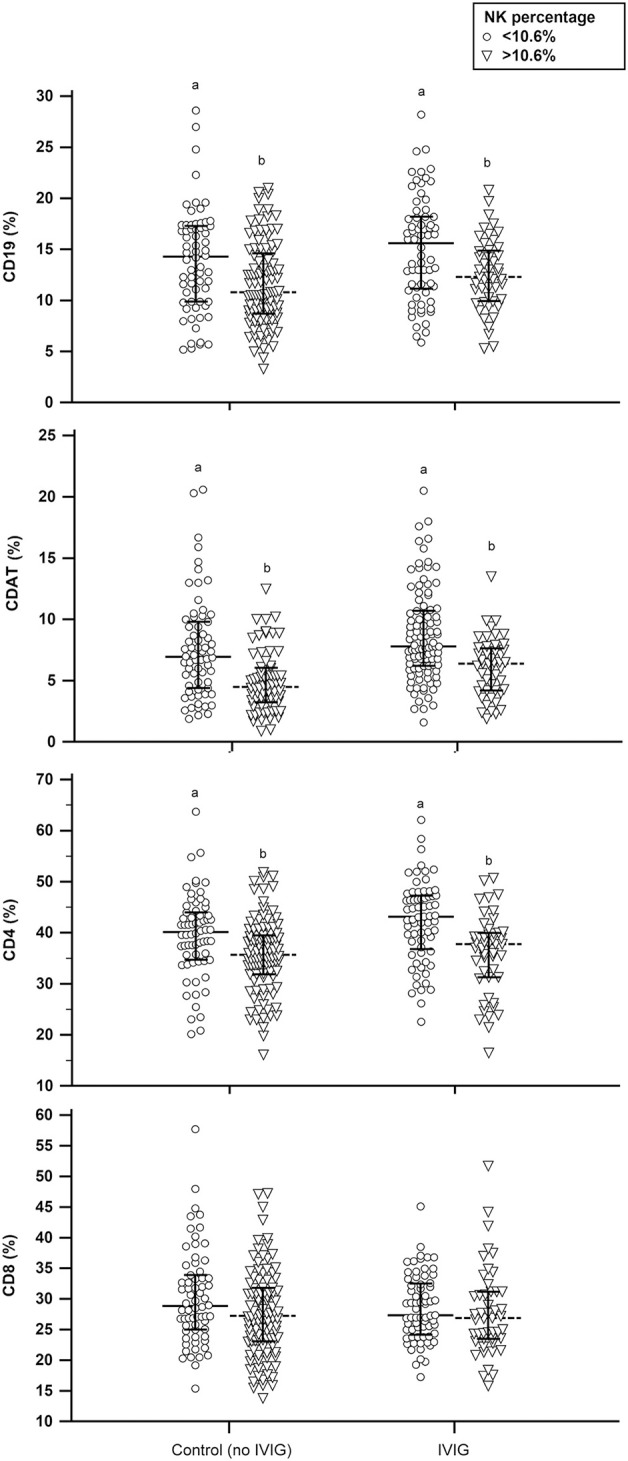
The distribution of peripheral mononuclear cell profiles between high and low percentage of CD56+CD16+ NK cells in intravenous immunoglobulin (IVIG) and non-IVIG groups. Different letters in the same subset figure indicate a significant difference, *P* < 0.01, using Mann-Whitney *U*-test.

None of the parameters in the peripheral mononuclear profile were found to be independent. Therefore, we compared each parameter of peripheral mononuclear cells based on the NK percentage. The patients with lower NK percentages (≤10.6%) had higher levels of CD19 cells, CDAT cells, and CD4 cells (Figure 4). In contrast, the levels of cytotoxic CD8 cells were similar between low and high NK groups (Figure 4). To determine the independence of the parameters in the peripheral mononuclear profile, we used multivariable logistic regression model for further analysis. The results indicated that the CD56^+^CD16^+^ NK cell percentage was the sole factor relevant to the ART outcome. The adjusted odds ratio for CD56^+^CD16^+^ NK cell percentage to correlated with pregnancy outcome is 1.061 (95% CI: 1.011–1.115), *P* = 0.017.

### A Percentage of ≤ 10.6% for Peripheral NK Cells in the Early Follicle Phase Is an Indicator for IVIG Treatment

All patients were divided into four subgroups according to the percentage of peripheral CD56^+^CD16^+^ NK cells in the early follicular phase: (1) IVIG group with >10.6% of CD56^+^CD16^+^ NK cells, (2) IVIG group with ≤ 10.6% of CD56^+^CD16^+^ NK cells, (3) non-IVIG group with >10.6% of CD56^+^CD16^+^ NK cells, and (4) non-IVIG group with ≤ 10.6% of CD56^+^CD16^+^ NK cells. In the non-IVIG groups, patients with ≤ 10.6% of CD56^+^CD16^+^ NK cells showed significantly lower implantation and pregnancy rates (12.3 and 30.3%, respectively) than patients with >10.6% of CD56^+^CD16^+^ NK cells (24.9 and 48.0%, respectively). The implantation, pregnancy and live birth rates in the IVIG group with a ≤ 10.6% NK percentage (27.5, 57.4, and 45.6%, respectively) were significantly higher when compared with the non-IVIG group with a ≤ 10.6% NK percentage (12.3, 30.3, and 22.7%, respectively). The benefit of IVIG treatment on implantation (25.0 vs. 24.9%) and pregnancy (61.7 vs. 48.0%) rates in the group with a >10.6% NK percentage was not significantly different from the non-IVIG group with a >10.6 NK percentage (Table 3). IVIG improved all outcome measures in those with NK ≤ 10.6%.

**Table 3:** Comparison of pregnancy outcomes of IVIG treatment between low and high NK cell percentages.

**(Reference range)**	**Low NK percentage (≤10.6%)**		**High NK percentage (>10.6%)**	
**Groups**	**Non-IVIG**	**IVIG**	**Non-IVIG**	**IVIG**
Cycles	66	68	102	47
Age (years)	36.1 ± 3.7	34.8 ± 4.4	36.7 ± 4.8	36.3 ± 5.1
Oocyte number	13.7 ± 9.4	14.0 ± 8.6	15.3 ± 9.6	17.2 ± 12.8
MII number	10.6 ± 7.7	11.3 ± 7.2	12.5 ± 8.0	13.8 ± 10.0
Fertilized embryo number	8.6 ± 9.2	9.2 ± 6.0	10.0 ± 6.9	11.0 ± 8.1
High qualified embryo rate	73.3 ± 14.9	70.3 ± 12.6	71.3 ± 13.5	69.7 ± 12.7
Transferred embryos	3.2 ± 0.8	3.6 ± 0.7	3.3 ± 0.8	3.5 ± 0.8
Implantation rate (%)	12.3 (26/212)^a^	27.5 (67/244)^c^	24.9 (85/341)	25.0 (41/164)
Pregnancy rate (%)	30.3 (20/66)^b^	57.4 (39/68)^d^	48.0 (49/102)	61.7 (29/47)
Live birth rate (%)	22.7 (15/66)	45.6 (31/68)^e^	35.3 (36/102)	40.4 (19/47)
Abortion rate (%)	25.0 (5/20)	17.9 (7/39)	24.5 (12/49)	34.5 (10/29)
Fetal body weight (gm)	2,939 ± 611	2,480 ± 678	2,625 ± 613	2,504 ± 527
Gestational age of delivery (weeks)	37.6 ± 2.8	36.9 ± 2.7	36.5 ± 2.8	35.6 ± 2.0

*The cutoff value of NK cell percentage (10.6 %) is selected by receiver operating characteristics curve analysis*.

*The data are presented with mean± standard deviation (SD) or percentage (%)*.

a P = 0.0003,

b *P = 0.023 compared with Non-IVIG group combined with high NK percentage (>10.6%) by X^2^ test*.

c P < 0.001,

d P = 0.002,

e *P = 0.005 compared with Non-IVIG group with low NK percentage (≤10.6%) by X^2^ test*.

After combining the patients and then subdividing into low NK and high NK groups, the patients in the high NK group showed a trend toward a higher implantation rate [20.4% (93/456) vs. 25.0% (126/505); *P* = 0.052] and abortion rate [20.3% (12/59) vs. 28.2% (22/78); *P* = 0.073] when compared with the low NK group.

## Discussion

In the present study, we found that a decreased percentage of peripheral CD56^+^CD16^+^ NK cells in the early follicle phase was significantly associated with low pregnancy rates using a logistic regression analysis. Furthermore, we demonstrated that an IVIG infusion was beneficial for implantation rates, pregnancy rates, and live-birth rates in RIF patients with a CD56^+^CD16^+^ NK cell population ≤ 0.6%. These data suggest that the peripheral blood CD56^+^CD16^+^ NK cell levels in the early follicle phase can be used to select patients who will benefit from IVIG.

A recent meta-analysis reported that the use of IVIG was beneficial for ART cycles in women with abnormal or elevated natural killer (NK) cells in the luteal phase; however, the strength of this evidence is poor (21). In this study, we examined peripheral monocytes in the early follicle phase rather than the luteal phase in order to merge the blood tests for ovarian reserves and peripheral mononuclear profiles for patients' convenience. Furthermore, this enabled us to merge the IVIG and IVF stimulation protocols. In addition, an early blood collection extends the available time for the patient to decide to undergo the rather costly IVIG treatment. Therefore, clinicians should balance efficiency vs. cost when deciding to treat certain conditions with IVIG. Appropriate patient selection and criteria for peripheral NK cell populations are crucial factors that determine the success of IVIG treatment.

The NK cells in the follicle and luteal phases may play different roles in implantation. During menstrual cycle, the percentage of peripheral blood NK cells (22) and the total number of peripheral NK-lymphocytes (23) significantly increased from the early follicular to luteal phase. However, Souza et al. (23) reported that NK cytotoxicity in the luteal phase was significantly reduced when compared with the follicular phase (*P* < 0.0001) in healthy women (24). Indeed, *in vitro* NK lymphocytes can differentiate into cells with NK1 (Th1) or NK2 (Th2) phenotypes similar to those of helper T lymphocytes (25). A shift in NK-lymphocytes toward a “Th2-type”-like response is only present during pregnancy and not in the luteal phase of the ovarian cycle. Thus, the NK cell and lymphocyte response shifts away from a type 1 immune response during pregnancy (26). Bouman et al. reported that monocytes may not be activated and are more sensitive in the luteal phase, whereas these cells are suppressed and less sensitive in the follicular phase (27). It has been suggested that a follicular ovarian factor exists that is capable of suppressing the non-specific immune system during the follicular phase (28, 29). Once this factor disappears (in the luteal phase or during pregnancy), the non-specific immune system is no longer suppressed and appears to be “activated and more sensitive”; thus, the specific immune system shifts toward a Th2 response (23, 30).

We suggest that NK cells in the early follicle may be an immune suppressing factor. Therefore, a lower NK population may reflect an unbalanced immune system, which leads to a specific immune system shift in the luteal phase after hormone stimulation. Although NK cells in the luteal phase were not examined in our study, we predict that the NK cells in the luteal phase of RIF patients were elevated, which further impaired embryo implantation. The physiological meaning of this phenomenon may be the preparation of the maternal immune system for potential implantation of the semi-allogenic blastocyst. However, further investigation is needed to confirm the occurrence of this phenomenon.

The uterine NK (uNK) cells are dominant and increase in absolute numbers in the decidua and to remodel the uterine arteries during pregnancy (14, 31, 32). The uNK cells are increase in the invading trophoblast and probably contribute to implantation (14). The uNK cells have been also described that their number increasing in the proliferative phase and reaching the maximal level in the late secretory phase during the menstrual cycle. These uNK cell numeric variations have been correlated to hormone-induced decasualization (30). The origin of uNK cells are presently unknown, and it is still debated whether they arise from NK cell progenitors present in the uterus prior to pregnancy, or are recruited from peripheral NK cell populations (30, 33). Carlino et al. suggest that peripheral NK cell recruitment to the uterus contributes to the accumulation of NK cells during early pregnancy and that progesterone plays a crucial role in this event (34).

The low NK population in the early follicle phase may reflect a lower recruitment of NK to the endometrium. The mechanisms controlling the accumulation of NK cells in the endometrium remain largely unknown. The propensity of NK cells to move into the decidua has been observed in all species investigated to date, suggesting a significant role for NK cells in normal pregnancy (35). Santillán et al. reported a positive correlation between blood and endometrial CD56^+^ NK cells (36), and Park et al. showed a correlation between the numbers of peripheral blood NK cells and endometrial NK cells from decidual tissue (37). Hanna et al. indicated that CD16^−^ NK cells are attracted from the peripheral blood to the decidua via CXCR4 and CXCL12 interactions; thus, the composition of the peripheral lymphocyte population is likely the key to a proper fetomaternal immune tolerance (38). In mouse models, decidual NK cells are recruited from peripheral sites rather than created from self-renewal processes in the uterine mucosa (39). Lee et al. reported that the increase of peripheral blood NK cells in the luteal phase may contribute to the recruitment of uterine NK cells from peripheral blood (22). Furthermore, Okitsu et al. used an autologous PBMC intrauterine administration to effectively improve embryo implantation in RIF patients (40). These data suggest that peripheral CD56^+^CD16^+^ NK cells are recruited into endometrium prior to embryo implantation. The results of our current study suggest that RIF women with low peripheral blood CD56^+^CD16^+^ NK cells in the early follicular phase may reflect an insufficiency of endometrial CD56^+^CD16^+^ NK cell recruitment and a defective microenvironment for embryo implantation.

However, previously published reports showed that a significantly elevated peripheral blood NK cell percentage in the luteal phase impaired female reproductive function (18, 41, 42). An appropriate NK population or recruitment in the luteal phase is very important. Our observations suggest that the percentage of peripheral NK cells in the early follicular phase also plays an important role in the regulation and recruitment of endometrial NK cells.

In our study, we found that the immune cell population in the early follicle phase between different menstrual cycles was similar. We collected samples from two separate no-stimulation cycles in a portion of RIF patients, and the peripheral mononuclear cell profiles (NK, CD19, or CD-AT) were not significantly different (data not shown). Therefore, the peripheral monocyte profile, especially the CD56^+^CD16^+^ NK cell population in the early follicle of RIF patients, may accurately reflect an immune unbalance associated with endometrial receptivity or fetomaternal immune tolerance. The chance of successful implantation in these patients could be strengthened by IVIG treatment.

In the present study, the reduced level of CD56^+^CD16^+^ NK cells was accompanied by an elevated percentage of CD4, CD-AT, and CD19 cells. These data suggest that in addition to CD56^+^CD16^+^ NK cells, T cells or B cells may represent an imbalance of innate and adaptive immune system function. The total number of monocytes was significantly lower in the follicular phase when compared with luteal phase (22, 43) and the changes may be connected to the immunobiology of implantation (22). Furthermore, the effect of different T regulatory cells (subsets of CD4^+^ T cells) (44) or CD-AT cells (45) correlated with ART outcome and implantation failure.

The strength of this study is the recruitment of a population of RIF patients receiving IVIG treatment to confirm the logistic regression analysis results from non-IVIG group and the benefit of IVIG treatment. The effect of IVIG infusion is prominent in patients with a low percentage of CD56^+^CD16^+^ NK cells when compared with patients who have a high NK percentage. NK cells are immune cells that can be distinguished by CD56 and CD16 surface antigen expression. In this study, only the CD56^+^CD16^+^ NK cells in the PBMC profile were detected because they represent the majority of all NK cells in the blood (46). NK cells are capable of binding to immune-complexed IgG via CD16-Fc gamma RIIIA molecules on the surface (47). NK cells produce cytokines that have pro-inflammatory and immunosuppressive effects [e.g., IFN-γ, tumor necrosis factor-α (TNF-α), and interleukin (IL)-10] and growth factors, such as granulocyte macrophage colony-stimulating factor (GM-CSF) and granulocyte colony-stimulating factor (G-CSF) (48, 49). Stimulation of CD16 on NK cells results in the production of cytokines, such as IFN-γ, TNF-α, and GM-CSF (47). IVIG induces antibody-dependent cellular cytotoxicity (ADCC) of mature dendritic cells (DCs) by NK cells, which downsizes the antigen-presenting pool and inhibits T-cell priming. By influencing the interaction between DCs and NK cells, IVIG modulates the ability of the innate immune system to trigger T-cell activation. This represents a mechanism that can “cool down” the immune system during times of activation (50). Several recent observations have emphasized the effects of IVIG therapy on a variety of cells from the innate and adaptive aspects of the immune system, including CD56^+^CD16^+^ NK cells, and various subsets of T cells and B cells (51). The results of the present study are in agreement with previous reports that show an association between unexplained RIF and immune imbalance in peripheral blood mononuclear cell profiles and the ability of IVIG to act as an immune modulator to enhance embryo implantation.

The limitation of this study was the lack of PBMC profiles from a fertile control group to compare with RIF groups. Further studies are needed to determine the NK profile after IVIG treatment. To confirm the importance of peripheral NK cells and monocytes in implantation, we have designed future studies to assess the PBMC population and cytokine expression in the early follicular phase without stimulation and the luteal phase after IVIG treatment in RIF patients.

In conclusion, we are the first to report that the peripheral CD56^+^CD16^+^ NK cell population in the early follicular phase is associated with IVIG outcomes in RIF patients. For RIF patients with a CD56^+^CD16^+^ NK cell population ≤10.6%, the implantation potential from IVF cycles is significantly lower than patients with NK cells >10.6%. In addition, IVIG treatment may be beneficial for these patients.

## Data Availability Statement

All datasets generated for this study are included in the article/supplementary material.

## Ethics Statement

The studies involving human participants were reviewed and approved by Institute of Review Board of the Chung Shan Medical University Hospital. The patients/participants provided their written informed consent to participate in this study.

## Author Contributions

Y-KH and H-HC were involved in conception and design of the study, performing experiments, acquisition of data, analysis and interpretation of data, and drafting of the article. T-HL and M-SL were involved in the analysis and interpretation of data, critical revision of the article, and final approval of the article. C-CH, C-IL, and P-YL was involved in the analysis and interpretation of data.

### Conflict of Interest

The authors declare that the research was conducted in the absence of any commercial or financial relationships that could be construed as a potential conflict of interest.
